# Evaluation of Humoral Immunity to *Mycobacterium tuberculosis*-Specific Antigens for Correlation with Clinical Status and Effective Vaccine Development

**DOI:** 10.1155/2015/527395

**Published:** 2015-10-19

**Authors:** Mamiko Niki, Maho Suzukawa, Shunsuke Akashi, Hideaki Nagai, Ken Ohta, Manabu Inoue, Makoto Niki, Yukihiro Kaneko, Kozo Morimoto, Atsuyuki Kurashima, Seigo Kitada, Sohkichi Matsumoto, Koichi Suzuki, Yoshihiko Hoshino

**Affiliations:** ^1^Department of Bacteriology, Osaka City University Graduate School of Medicine, Abeno, Osaka 545-8585, Japan; ^2^National Hospital Organization, National Tokyo Hospital, Takeoka, Kiyose, Tokyo 204-8585, Japan; ^3^Division of Respiratory Medicine, Fukujuji Hospital, Japan Anti-Tuberculosis Association, Matsuyama, Kiyose, Tokyo 204-8522, Japan; ^4^National Hospital Organization, National Toneyama Hospital, Toneyama, Toyonaka, Osaka 560-8552, Japan; ^5^Department of Infectious Disease Control and International Medicine, Niigata University Graduate School of Medical and Dental Sciences, Niigata 951-8510, Japan; ^6^Department of Mycobacteriology, Leprosy Research Center, National Institute of Infectious Diseases, Aoba, Higashimurayama, Tokyo 189-0002, Japan

## Abstract

Although tuberculosis remains a major global health problem, Bacille Calmette-Guérin (BCG) is the only available vaccine. However, BCG has limited applications, and a more effective vaccine is needed. Cellular mediated immunity (CMI) is thought to be the most important immune response for protection against *Mycobacterium tuberculosis* (Mtb). However, the recent failure of a clinical trial for a booster BCG vaccine and increasing evidence of antibody-mediated immunity prompted us to evaluate humoral immunity to Mtb-specific antigens. Using Enzyme-Linked ImmunoSpot and Enzyme-Linked ImmunoSorbent Assays, we observed less correlation of both CMI and IgG titers with patient clinical status, including serum concentration of C reactive protein. However, IgA titers against Mtb were significantly correlated with clinical status, suggesting that specific IgA antibodies protect against Mtb proliferation. In addition, in some cases, IgA antibody titers were significantly associated with the serum concentration of total albumin, which supports the idea that humoral immunity can be influenced by the nutritional status. Based on these observations, we propose that the induction of humoral immunity should be included as an option in TB vaccine development strategies.

## 1. Introduction

Tuberculosis (TB) remains a leading cause of death in many regions of the world and one-third of the world's population is thought to be asymptomatic carriers of the causative agent,* Mycobacterium tuberculosis* (Mtb). The World Health Organization estimates that approximately 8.6 million people contracted the disease and 1.3 million died in 2012 [1]. Bacillus Calmete-Guerin (BCG), the only approved and available TB vaccine, is made using an attenuated strain of* Mycobacterium bovis*, a relative of Mtb [2]. The primary use of the BCG vaccine is the immunization of children in areas with a high prevalence of TB. However, BCG is considered an inadequate vaccine because it offers no protection for adults [3]. A novel approach is needed to create a more effective vaccine.

Several novel approaches to vaccine production are in development. Recent progress in methods of vaccine development has been covered in several excellent reviews and will not be addressed here [4, 5]. The majority of these candidate vaccines focus on improving cell-mediated immunity (CMI) to Mtb by modifying the current BCG platform or boosting BCG with a different format, and some candidate vaccines have shown promise in mice and/or nonhuman primates [6, 7]. Modified Vaccinia Ankara (MVA) 85A is one of the more advanced vaccine candidates that use BCG with a booster of Mtb major secreted antigen complex 85A (Ag85A) [8], which boosts BCG-primed Th1 immune responses. It was expected that MVA85A would reduce the incidence of TB cases in an endemic area (i.e., South Africa) by 60%. However, the phase 2b clinical trial found that MVA85A conferred no detectable improvement against Mtb infection [9].

Although CMI has been established as a major component in the control of mycobacterial infections, serology analyses indicate that Mtb infection also induces humoral immune responses against various mycobacterial antigens [10]. In addition, BCG vaccination can induce antibody responses to several mycobacterial antigens [11–13], which elicits both innate and cell-mediated immunity against mycobacteria [13]. Humoral immunity could modify the fate of intracellular bacteria through mechanisms ranging from simple opsonization to complicated FcR activation [14].

The consensus for many years was that the control of vigorous granuloma formation following infection by pathogenic fungi (i.e., intracellular pathogens) was achieved by activating CMI, while humoral immunity was believed to have no role in protection. However, recent studies using hybridoma technology found protective monoclonal antibodies against numerous pathological fungi [15]. Two ongoing vaccine trials for the fungus* Candida albicans*, a major human pathogen, are expected to transduce protection by inducing antibody-mediated immunity [16].

These studies suggest that humoral immunity might also play an important role in protection against Mtb infection, a function which could be used to develop a new technology for more effective vaccine development. To evaluate the feasibility of using humoral immunity against mycobacterial antigens for effective vaccine development, we compared IgG and IgA antibody titers using a variety of clinical and immunological parameters. The results suggest that IgA antibodies against Mtb components would make suitable Mtb vaccine candidates.

## 2. Subjects and Methods

### 2.1. Participants

Patients of National Tokyo Hospital, Tokyo, Japan, were consecutively enrolled in the study, after giving informed consent, from May 2010 through May 2011. A total of 190 Japanese patients (age: 60.1 ± 19.6 yr, male: 71.1%) were recruited. The following information was obtained from all patients at the time of enrollment: history of prior TB disease, work history in any healthcare setting or recent exposure to a patient with active TB, and other TB risk factors such as having immunodeficiency disorders or taking immunosuppressive drugs [17]. We used the same inclusion/exclusion criteria as in a previous study [18]. Information on previous medical history, clinical signs and symptoms, and radiological and microbiological data including the values of serum C reactive protein (CRP) and the concentration of total albumin were also collected. The patients were then divided into three categories: (1) Active disease: patients having positive TB symptom(s) and positive smear results and/or positive demonstration of Mtb in culture; (2) Past disease: previously diagnosed with TB, treated and currently free from symptom(s); and (3) Latent TB infection (LTBI): no symptoms with normal chest X-ray, but having positive results from an interferon gamma release assay (IGRA). Among the 190 patients recruited, 88 (46%) were classified as “Active disease,” 84 (45%) as “Past disease,” and 18 (9%) as LTBI. We enrolled age- and gender-matched patients with respiratory disease who were confirmed not to have any mycobacterial diseases as negative controls. All medical, radiological, and microbiological information was collected to confirm their eligibility. A total of 77 Japanese adults (age: 63.9 ± 10.7 yr, male: 71.4%) were recruited as a control group (Table 1). In Japan, more than 90% of the population has been vaccinated with BCG since 1929, suggesting that most of the control group have received the BCG vaccine. The research protocol was approved by the Institutional Review Boards of Osaka City University Graduate School of Medicine, Osaka, Japan, National Tokyo Hospital, Tokyo, Japan, Fukujuji Hospital, Tokyo, Japan, and National Toneyama Hospital, Osaka, Japan, and by the Research Ethics Committee of the National Institute of Infectious Disease, Tokyo, Japan.

### 2.2. Evaluation of Clinical Status

The clinical status of patients with active disease was evaluated as previously described with modification [19] (see Supplemental Table  1 in Supplementary Material available online at http://dx.doi.org/10.1155/2015/527395). (1) “Smear at entry” (entry = point of diagnosis before treatment): sputum smear taken at entry was stained and inspected by microscopy. The severity was subdivided as 0 (no acid fast bacilli (AFB) on smear), ± (1-2 AFB per 300 field), 1+ (1–9 AFB per 100 field), 2+ (more than 10 AFB per 100 field), and 3+ (more than 10 AFB per field). (2) “Positive conversion time”: duration (weeks) between entry and positive MGIT results. (3) Duration of culture negative: time (days) from initiation of treatment to negative smear results (= 0 AFB per field) by sputum microscopy. (4) Several routine laboratory tests including serum concentration of “C reactive protein (CRP) at entry,” (total) “Albumin at entry,” and “CRP after 60 days of treatment” were simultaneously performed. (5) Severity of chest radiography at entry: “The Japanese Society for Tuberculosis Classification” (1959) was applied [20]. Briefly, tuberculosis lesions are classified by chest X-ray findings as type (cavity) and extent. “X-ray type (cavity)” was subdivided from III to I (III: no cavity, II: morbid foci other than I, and I: widespread cavities) and “X-ray extent” from 1 to 3 (1: minimal, 2: moderate, and 3: severe). These markers and classifications were chosen because serum albumin levels reaction to the tuberculin skin test, the Gaffky scale, and negative conversion time are significantly correlated with both type and extent [20]. If patients contracted hilar glandular tuberculosis and/or tuberculosis lymphadenopathy, the evaluation of (1), (2), and (3) was excluded and that of (4) and (5) was included for the analysis because bacterial presentation in the sputum is rare.

### 2.3. QuantiFERON-TB Gold In-Tube (QFT-GIT) Assay

The QFT-GIT assay was performed using fresh whole blood in accordance with the manufacturer's instructions (Cellestis, Chadstone, Australia). The results were interpreted with software provided by Cellestis. The antigen used in this assay is the 6 kDa early secreted antigenic target from* M. tuberculosis *(ESAT-6), the 10 kDa culture filtrate protein (CFP-10), and TB7.7. However, there was no response to TB7.7 in Japanese patients [17]. Results were scored as “positive” when the IFN-*γ* concentration in the tube with TB-specific antigen was >0.35 IU/mL, after subtracting the value of the nil control, and at least >25% of the negative control value. When the net IFN-*γ* response was <0.35 IU/mL for the antigens and the response to the mitogen-positive control was >0.5 IU/mL, the response was considered “negative” [17, 19]. Stimulation with mitogen induced more than 0.5 IU/mL in all subjects, supporting the contention that immunosuppressed participants were not included in the study (data not shown).

### 2.4. ELISPOT Assay

The IFN-*γ* ELISPOT assay was performed as previously described [17]. Briefly, peripheral blood mononuclear cells (PBMCs) were seeded in precoated IFN-*γ* ELISPOT plates (Becton, Dickinson and Company, Franklin Lakes, NJ, USA) with 2.5 × 10^5^ cells per well in AIM-V medium (GIBCO) and incubated with a protein (10 *μ*M) of each antigen at 37°C in 5% CO_2_ for 16 hr. A negative control (no mitogen or antigen) and a positive control (phytohemagglutinin, PHA, 5 *μ*g/mL) were also included. After incubation, the wells were washed and developed with a conjugate against the antibody used and an enzyme substrate. Spot-forming units were counted using a KS ELISPOT imaging system (Carl Zeiss, Hallbergmoos, Germany) and labeled spot-forming cells (SFC). ELISPOT results were interpreted according to the following criteria: the test result was positive when (1) the negative control had 0–5 spots and (2) the spot count (negative control spot count) was greater than six. The test result was negative when the above criteria were not met and the positive control was valid (≥20) [17, 21]. Data were plotted as the numbers of SFC/1 × 10^6^ cells.

### 2.5. ELISA Assay

Concentrations of IgG and IgA antibodies against Mtb were determined by ELISA using recombinant proteins as previously described, with modification [22]. Ninety-six well microplates (Sumilon Type H, LMS, Tokyo, Japan) were coated with each recombinant antigen in bicarbonate buffer, pH 9.6 overnight at 4°C (Supplemental Table  2). The plates were blocked with phosphate buffered saline (PBS) containing 0.05% Tween 20 and 5% skim milk for 12 hr at 4°C and washed four times with PBS containing 0.05% Tween 20. Human serum samples diluted 1 : 200 (for IgG) or 1 : 100 (for IgA) in PBS containing 0.05% Tween 20 and 0.5% skim milk were then added in duplicate (IgG) or triplicate (IgA) to the antigen-coated wells and incubated for 12 hr at 4°C. After washing the wells, HRP-conjugated antihuman IgG or IgA antibodies were added at a 1 : 2000 or 1 : 1000 dilution, respectively. Following a one hr incubation at 37°C, the plates were washed four times before 100 *μ*L of SureBlue reserve-TMB was added to each well. The reactions were stopped after 10 min by adding 50 *μ*L of 0.1 M HCl, and absorbance was measured at 450 nm using a Multiskan Spectrophotometer (Thermo Fisher Scientific, Yokohama, Japan). The results of the IgG-ELISA were expressed as absorbance at 450 nm, whereas results of the IgA-ELISA were expressed as ELISA-Index, *S*/(*B* + 3SD) [23, 24], where *S* is the average OD value of the duplicate test samples and *B* + 3SD corresponds to the average OD value of the duplicate negative controls (*B*) plus three times the standard deviation (SD).

### 2.6. Reagents and Recombinant Protein Preparation

pET-21b, pET-22b, and Bugbuster HT were obtained from Novagen (Darmstadt, Germany);* Escherichia coli* BL21 (DE3) cells were from Toyobo (Osaka, Japan); Lowenstein-Jensen Luria-Bertani medium and carbenicillin were from Sigma (St. Louis, MO, USA); isopropyl-1-thio-beta-d-galactopyranoside and Ni-NTA agarose were from Qiagen (Gaithersburg, MD, USA); skim milk was from Morinaga (Tokyo, Japan); horseradish peroxidase-conjugated antihuman IgG or IgA antibodies and Envision kits were from Dako (Carpinteria, CA, USA); SureBlue reserve TMB microwell peroxidase substrate was from KPL (Gaithersburg, MD, USA); and monoclonal Acr antibody was from HyTest (Turku, Finland).

A pET-21b or pET-22b-based vector expressing 16 kDa *α*-crystallin homolog (Acr, 16 kDa protein, hspX or TB16.3: Rv2031c), Ag85A (Rv3804c), CFP-10 (Rv3874), ESAT-6 (Rv3875), heparin-binding haemagglutinin adhesin (HBHA: Rv0475), heat-stress-induced ribosome binding protein A (HrpA, 20 kDa protein, Acr2 or hsp20: Rv0251c), and mycobacterial DNA-binding protein 1 (MDP1: Rv2986c) were produced by a PCR-based approach using a bacterial chromosome. Each PCR product containing coding regions was designed to allow expression of C-terminal, 6 histidine-tagged variants of the recombinant proteins following ligation into pET-21b. After construction, expression vectors were confirmed by DNA sequencing. Recombinant Mtb proteins were purified using Ni-NTA columns (1 mL bed volume, GE Healthcare, Piscataway, NJ, USA) according to the manufacturer's instructions. Purified proteins were used as mycobacterial antigens for ELISAs and ELISPOT assays.

### 2.7. Statistical Analysis

The Mann-Whitney *U* test was used to compare IgG and IgA levels between two independent groups, whereas one-way ANOVA was used for the comparison of three or more unmatched groups. Spearman's rank correlation coefficient was used to determine the correlation between ELISA values and the severity of clinical status values. Pairwise comparisons were made between areas under the receiver operating characteristic curve (AUROC) for the categorized groups. Optimal cut-off values were chosen when Youden's index (sensitivity and specificity − 1) was maximal. All analyses were performed using online statistics calculators (http://www.socscistatistics.com/tests/Default.aspx, http://vassarstats.net/index.html, http://molpath.charite.de/cutoff/index.jsp). The threshold of significance was set at *P* < 0.05.

## 3. Results

### 3.1. Antibody Titers in Active Disease, Past Disease, LTBI Cases, and Controls

**Figure 1:**
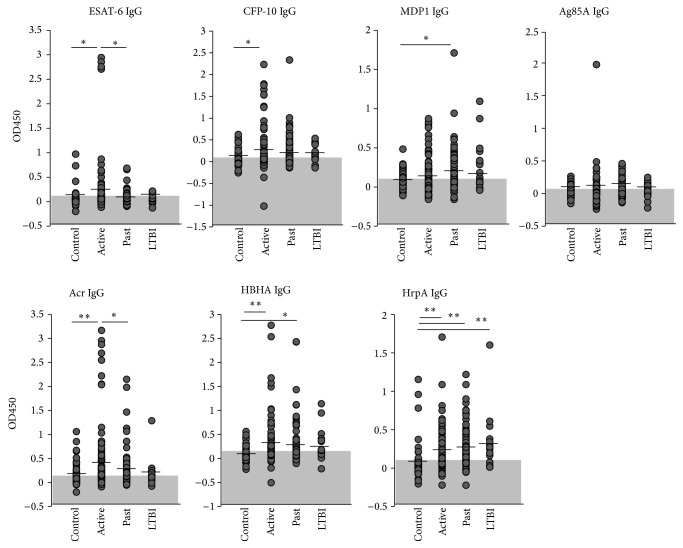
IgG responses to Mtb antigens. The levels of serum IgG against 7 antigens (ESAT-6, CFP-10, MDP1, Ag85A, Acr, HBHA, and HrpA) in active disease (labeled as “Active”), past disease (“Past”), latent TB infection (“LTBI”), and controls (“Control”) were analyzed by ELISA. Data shown are the average of triplicate experiments. Shaded areas: areas under cut-off values, vertical lines: mean values, ^∗^ *P* < 0.05, ^∗∗^ *P* < 0.01.

**Figure 2:**
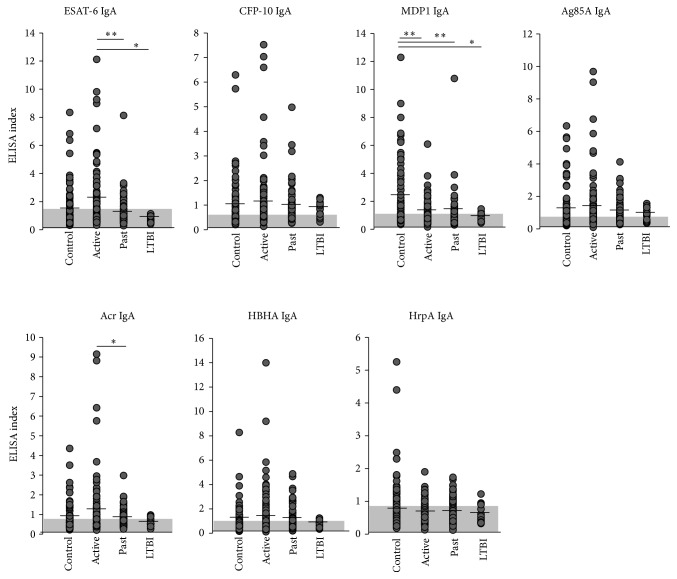
IgA responses to Mtb antigens. The levels of serum IgA against 7 antigens (ESAT-6, CFP-10, MDP1, Ag85A, Acr, HBHA, and HrpA) in active disease (labeled as “Active”), past disease (“Past”), latent TB infection (“LTBI”), and controls (“Control”) were analyzed by ELISA. Data shown are the average of triplicate experiments. Shaded areas: areas under cut-off values, vertical lines: mean values, ^∗^ *P* < 0.05, ^∗∗^ *P* < 0.01.

Titers of IgG and IgA antibodies against recombinant mycobacterial antigens were measured separately in the sera of “Active disease” (*n* = 88), “Past disease” (*n* = 84), LTBI (*n* = 18) cases, and control (*n* = 77). We chose mycobacteria-specific antigens based on (1) proteome analysis of antibody responses to Mtb from active TB patients (included Acr, Ag85, CFP-10, and HrpA) [25, 26]; (2)* in silico* studies performed to identify TB vaccine candidates (included Acr, Ag85, and ESAT-6) [27]; and (3) publications identifying HBHA and MDP1 as mycobacteria-specific antigens [22, 28, 29]. Active disease patients had higher levels of IgG antibodies against all antigens tested compared to controls. In particular, significant increases in serum IgG levels were observed against ESAT-6, CFP-10, Acr, HBHA, and HrpA (Figure 1). Titers of past disease were lower than those of active disease except for MDP1, Ag85A, and HrpA (Figure 1). In contrast, IgA antibody titers for MDP1 and HrpA were higher in the control group than in the active disease group (Figure 2). The individual values of antibodies against the seven antigens were used to generate receiver operating characteristic curves (ROC). The Area under the ROC curves (AUROC) was calculated in both Tables 2 and 3.

### 3.2. ELISPOT and QFT Titers in Active Disease, Past Disease, and LTBI and Comparison between CMI Assays and Humoral ELISA Assays

CMI against various recombinant antigens was measured by ELISPOT assay for the active disease, past disease, and LTBI groups. Ninety-six participants (age: 58.6 ± 18.9 yr, male: 68.8%) were randomly chosen for the analysis of IFN-*γ* ELISPOT assay for ESAT-6 and CFP-10 antigens. A portion of the participants were also randomly recruited for the ELISPOT assay (MDP1: *n* = 37 (age: 55.9 ± 18.2 yr, male: 67.6%); Acr: *n* = 36 (age: 55.7 ± 18.4 yr, male: 66.7%); HBHA: *n* = 20 (age: 56.2 ± 17.7 yr, male: 100%); and HrpA: *n* = 29 (age: 56.7 ± 16.8 yr, male: 100%)).

**Figure 3:**
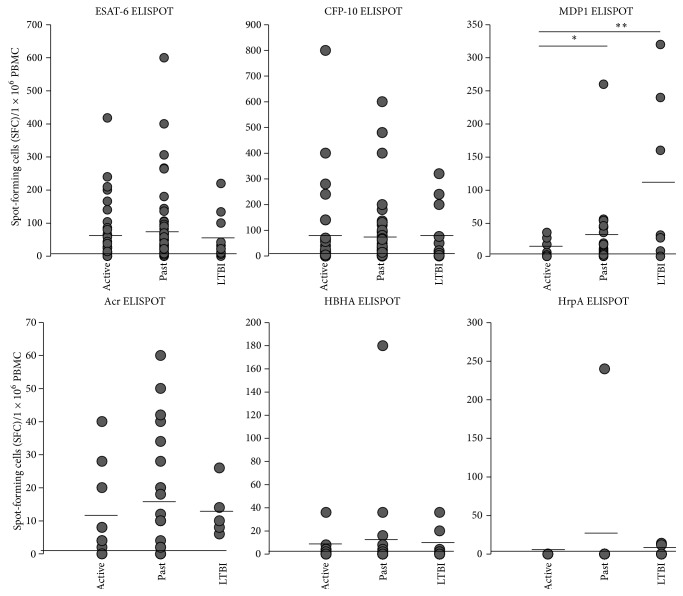
ELISPOT responses to Mtb antigens. ELISPOT SFCs against 6 antigens (ESAT-6, CFP-10, MDP1, Acr, HBHA, and HrpA) in active disease (labeled as “Active”), past disease (“Past”) and latent TB infection (“LTBI”). Data shown are the average of triplicate experiments. Vertical lines: mean values, ^∗^ *P* < 0.05, ^∗∗^ *P* < 0.01.

No significant difference was observed in CMI, except for MDP1, in which past disease and LTBI patients had significantly more SFCs than active disease patients (Figure 3). Stimulation with PHA induced more than 80 SFC per 1 × 10^6^ cells in all subjects (data not shown), indicating the lack of immunosuppressed participants in the study. When the results from CMI (ELISPOT) and humoral immunity (ELISA to IgG and IgA) were compared, there was no association between ELISPOT positivity and the values of IgG or IgA (Supplemental Figure  1). Also, there were no significant differences in the values of QFT-GIT assay among the active disease, past disease, and LTBI groups (Supplemental Figure  2).

### 3.3. “CRP at Entry” as a Surrogate Marker of Other Clinical Markers

**Figure 4:**
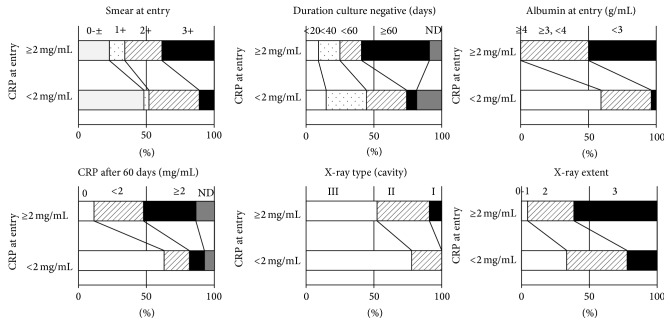
Clinical markers evaluated with “CRP at entry.” All markers were significantly associated with “CRP at entry.” “CRP at entry” was significantly associated with “Smear at entry” (*r* = 0.296, *P* < 0.05), “Duration of culture negative” (*r* = 0.391, *P* < 0.01), “Albumin at entry” (*r* = 0.687, *P* < 0.01), “CRP at 60 days” after treatment (*r* = 0.528, *P* < 0.01), X-ray type (cavity) (*r* = 0.271, *P* < 0.05), and X-ray extent (*r* = 0.445, *P* < 0.01).

We divided serum CRP values at entry into two categories: CRP < 2 mg/mL (negative or minimal inflammation) and CRP ≥ 2 mg/mL (intermediate or severe inflammation) because CRP is a more sensitive inflammation marker than erythrocyte sedimentation rate, another systemic inflammation marker. Other clinical markers were also divided into several categories (from negative to severe, Supplemental Table  1). All other clinical markers, such as “Smear at entry,” “Positive conversion time,” “Duration of culture negative,” “Albumin at entry,” “CRP at the time after 60 days,” “X-ray type (cavity),” and “X-ray extent” were evaluated with the scores of “CRP at entry” (Figure 4). The “Smear at entry” (*r* = 0.296, *P* < 0.05), “Duration of culture negative” (*r* = 0.391, *P* < 0.01), “Albumin at entry” (*r* = 0.687, *P* < 0.01), “CRP after 60 days” (*r* = 0.528, *P* < 0.01), “X-ray type (cavity)” (*r* = 0.271, *P* < 0.05), and “X-ray extent” (*r* = 0.445, *P* < 0.01) scores were significantly associated with those of “CRP at entry,” suggesting that “CRP at entry” could serve as a surrogate for other clinical markers. Only “Positive conversion time” was not associated with “CRP at entry” (*r* = 0.09, *P* = 0.45, data not shown).

### 3.4. Reverse Association between IgA Titers against HrpA and “CRP at Entry”

**Figure 5:**
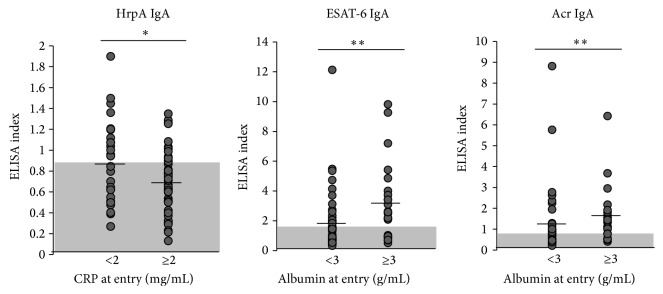
Association between immunological scores and clinical scores. HrpA IgA was negatively associated with “CRP at entry.” ESAT-6 IgA and Acr IgA were positively associated with “Albumin at entry.” Shaded areas: areas under cut-off values, vertical lines: mean values. ^∗^ *P* < 0.05, ^∗∗^ *P* < 0.01.

Finally, we compared the results of immunological scores and clinical scores, such as “Smear at entry,” “Positive conversion time,” “Duration of culture negative,” “CRP at entry,” “Albumin at entry,” “CRP at the time after 60 days,” “X-ray type (cavity),” and “X-ray extent.” There was no association among IgG antibodies. Among IgA antibodies, “CRP at entry” was significantly associated with HrpA IgA levels (*r* = −0.2505, *P* < 0.05); ESAT-6 IgA was significantly associated with “Albumin at entry” (*r* = 0.3304, *P* < 0.01); and Acr IgA was also associated with “Albumin at entry” (*r* = 0.3334, *P* < 0.01) (Figure 5). No association was found between immunological markers and clinical markers for other measured parameters (Supplemental Figures  3–10 and data not shown).

## 4. Discussion

We compared serum antibody titers and IGRA against components of Mtb to evaluate humoral and cell-mediated immunity for improvements in vaccine development. We also compared these data with several clinical indices to evaluate a possible link for disease progression. Some IgA titers were elevated in the controls and lower in the active disease group. “CRP at entry” was significantly associated with several other clinical parameters. IgA antibody levels against HrpA in active disease patient were significantly associated with the clinical inflammation status measured by “CRP at entry.” In addition, IgA antibodies against ESAT-6 and Acr were significantly associated with clinical nutrition status as measured by “Albumin at entry.” Notably, an inverse correlation was found between “CRP at entry” and the IgA titer for HrpA, and a positive correlation was revealed between “Albumin at entry” and IgA titers for ESAT-6 and Acr. These findings suggest that some IgA antibodies targeting mycobacterial antigens can protect against the bacterial expansion or replication and corresponding lung inflammation previously reported in murinestudies* in vivo* [23, 30–32] and that antibody production could be influenced by the nutritional status of the patients, as has been reported in many studies [33–36].

CMI induction of the Th1 response should be the center of the principal immunity to Mtb infection. However, a recent study revealed this is not always the case. Strong immunological pressure on microbes usually drives an antigen shift, whereas a whole genome analysis of human T cell epitopes of Mtb showed they are evolutionarily hyperconserved [37]. If humans eliminate Mtb primarily through CMI, bacterial antigens might be exposed by the high level of immunological pressure; therefore, the epitopes should be hypermutated as they are in other pathogens such as hepatitis B virus, hepatitis C virus, HIV, or influenza virus [38–41]. We also found that most CMI measured by IFN-*γ* ELISPOT showed no difference among active, past, and LTBI patients. In addition, the frequency of IL-17+ CD4 cells (Th17 cells) was increased in HBHA or MDP1 responded populations in active or past TB patients [28]. These observations led us to initiate the present study in order to examine the possibility of an additional immune response to mycobacterial infection.

Our study of cellular gene expression, derived by bronchoalveolar lavage (BAL) in active TB patients as well as normal volunteers using DNA microarray analysis, indicated a shift from a Th2 to Th1 phenotype in lung immune cells during the course of tuberculosis [42]. Most of the BAL cells from active patients showed a Th1 phenotype whereas all normal volunteers and some TB patients were Th2. Notably, the phenotype shifted from Th2 to Th1 in a patient during the course of the disease, supporting the premise that humoral immunity is predominant in the early stage of infection [16]. This observation suggests that humoral immunity might be an effective target for adjuvant enhancement in a new TB vaccine.

Recent studies have revealed that approximately 10% of bacterial proteomes could generate a human antibody response and a much smaller fraction of antigens (estimated as less than 1%) could be preferentially recognized by serum antibodies in active TB patients [25, 26]. We noted that the titers of some IgA antibodies were lower in active TB patients than in the controls, suggesting that BCG-induced humoral immunity to Mtb is maintained even after adolescence, but active disease can occur when the immunity diminishes. Several studies have also confirmed that vaccination with specific Mtb antigens such as Acr, Ag85, CFP-10, ESAT-6, or HBHA efficiently induces IgA and/or IgG antibodies as well as IFN-*γ* and other cytokines [35–40]. It is possible that DNA vaccination can induce humoral immunity in addition to CMI, which is important in protecting against the growth of Mtb. [30–32, 43–49].

There are many reports analyzing the level of IgG antibody titers in different clinical stages of TB [22, 50–52]. The major conclusion of these studies is that most IgG antibodies increase in the active phase and decline following treatment or during a LTBI, suggesting that the bacterial load is associated with the production of IgG and that the clinical cure lowers immunoglobulin levels. In this study, we confirmed that Mtb-specific IgG antibody levels are associated with bacterial load, because most IgG values were higher in active patients than past patients. However, we noted that IgA antibody titers of HrpA and MDP1 were quite different from those of IgG antibodies as they were higher in controls than in active patients. These results suggest that IgA levels toward some Mtb antigens are modified after infection, even if the patients had previously received a BCG vaccination as most Japanese should. Based on these findings, it can be hypothesized that a decline in IgA levels for these antigens, which is initially induced by BCG vaccination, might be related to bacterial growth. Although the DNA sequence of these antigens is almost homologous between BCG and Mtb (Supplemental Table  3), there may be a difference in protein structure and/or an amino acid sequence that is essential for the induction of humoral immune responses.

Both Acr and HrpA are mycobacterial heat shock proteins or chaperones that were revealed by a whole genome analysis of H37Rv [53]. Acr is a well characterized mycobacterial protein that possesses immune-dominancy [31, 32, 48, 54, 55]. Acr plays important roles during log phase growth and transition to the stationary phase of Mtb proliferation [56, 57]. Moreover, there are some studies concluding that monoclonal IgA antibody against Acr protects mycobacterial proliferation* in vivo*. Williams et al. compared the effect of TBA61 (anti-Acr monoclonal IgA) and TBA68 (IgG of the same epitope as TBA61) on Mtb growth and observed that IgA antibody contributed more to a reduction in the number of bacteria in mouse lung space [31]. López et al. reported a greater reduction of bacteria and pathological severity of the disease when using TBA 61 compared with TBA84 (IgA to PstS-1, 38 kDa secreted glycoprotein [58, 59]) [32]. Although these experiments were performed using mouse models, they suggested that humoral immunity could at least modify or reduce the growth of Mtb [31, 32]. HrpA was identified by Ohara et al. in 1997 [60] with a 30% homology to Acr, which increases to 40% when comparing Acr core residues [61]. It was later demonstrated that the expression of HrpA is upregulated by heat-shock, nitrate, or macrophage engulfment, suggesting that HrpA is one of the early immune targets of Mtb antigens [62]. We found that the IgA to Acr or HrpA was associated with some of the parameters used to measure the clinical status of active patients and that it could support inhibition of the mycobacterial burden.

We also revealed that “CRP at entry” is a good surrogate marker for other clinical markers, a finding which is consistent with reports of previous studies [63]. Hypoalbuminemia has emerged as an independent factor of poor prognosis in several studies [33–36]. In this study, IgA antibody titers generated by certain Mtb-specific antigens were significantly correlated with the scores of clinical markers such as CRP or albumin levels at first admission, which appears to be one of the host immune responses against Mtb. There was a significant association between IgA antibody titer and albumin. The importance of nutritional status for the clinical manifestation of TB has been evidenced by the fact that the incidence of TB is much higher in developing countries. This position is also supported by the correlations of serum IgA titers and higher albumin levels in the present study. The fact that humoral immunity had no association with CMI suggests that mycobacterial antigens influence humoral and cellular mediated immunity by different mechanisms. These findings suggest that induction of the IgA response could be a good strategy for Mtb vaccine design.

## 5. Conclusion

IgA antibody titers against several Mtb antigens, but not IgG antibodies nor CMI, significantly correlate with the clinical status of TB patients, raising the possibility that specific IgA antibodies protect against promotion of Mtb. These observations also suggest that induction of humoral immunity, especially for the IgA response, should be included as an option for TB vaccine strategies.

## Supplementary Material

Supplemental Table 1: Classification of various clinical laboratory test results that relate to the patients' general condition (serum albumin), inflammatory status (C-reactive protein level; CRP) and the disease status (smear at entry, positive conversion time, duration of culture negative, X-ray type and X-ray extent).
Supplemental Table 2: List of the amount of recombinant Mtb antigens used to evaluate the serum IgG and IgA levels by ELISA as described in Subjects and Methods 2.5.
Supplemental Table 3: Statistical analysis of the association between serum IgA levels and the levels of various clinical statuses classified in Supplemental Table 1.
Supplemental Figure legends
Supplemental Figure 1: Association between humoral IgG or IgA response and positivity of ELISPOT assays using the same antigens. There was no association between IgG or IgA values and ELISPOT positivity against the same antigens. Shaded areas: areas under cut-off values, vertical lines: mean values, +: ELISPOT positive and -: ELISPOT negative.
Supplemental Figure 2: Association of QFT-IT assay values in active disease, past disease and LTBI patients. There was no significant association in these patients. Vertical lines: mean values, values: concentration of IFN-γ (IU/ml).
Supplemental Figure 3: Association between humoral IgA responses and clinical scores measured by smear at entry. There was no association between IgA values and scores measured by smear at entry. Shaded areas: areas under cut-off values, vertical lines: mean values.
Supplemental Figure 4: Association between humoral IgA responses and clinical scores measured by positive conversion time (weeks). There was no association between IgA values and scores measured by positive conversion time. Shaded areas: areas under cut-off values, vertical lines: mean values, ND: not done.
Supplemental Figure 5: Association between humoral IgA responses and clinical scores measured by duration culture positive (days). There was no association between IgA values and scores measured by duration culture negative. Shaded areas: areas under cut-off values, vertical lines: mean values, ND: not done.
Supplemental Figure 6: Association between humoral IgA responses and clinical scores measured by CRP at entry (mg/ml). There was no association between IgA values and scores measured by CRP at entry. Shaded areas: areas under cut-off values, vertical lines: mean values, ND: not done.
Supplemental Figure 7: Association between humoral IgA responses and clinical scores measured by Albumin at entry (g/ml). There was no association between IgA values and scores measured by Albumin at entry. Shaded areas: areas under cut-off values, vertical lines: mean values, ND: not done.
Supplemental Figure 8: Association between humoral IgA responses and clinical scores measured by CRP after 60 days (mg/ml). There was no association between IgA values and scores measured by CRP after 60 days. Shaded areas: areas under cut-off values, vertical lines: mean values, ND: not done.
Supplemental Figure 9: Association between humoral IgA responses and clinical scores measured by X-ray type (cavity). There was no association between IgA values and scores measured by X-ray type (cavity). Shaded areas: areas under cut-off values, vertical lines: mean values.
Supplemental Figure 10: Association between humoral IgA responses and clinical scores measured by X-ray extent. There was no association between IgA values and scores measured by X-ray extent. Shaded areas: areas under cut-off values, vertical lines: mean values.

## Figures and Tables

**Table 1:** Characteristics of patients in this study.

	Active disease	Past disease	LTBI	Control
*n*	88	84	18	77
Age (mean ± SD)	59.9 ± 20.2	60.5 ± 19.6	58.8 ± 17.7	63.9 ± 10.7
Age range	21–97	19–92	21–87	35–87
Sex (male/female)	65/23	59/25	11/6	55/19
Positivity of QFT-IT	69.3%	59.5%	64.7% (100%^a^)	ND

^a^Patients were all positive for any IRGA (QFT-GIT and/or ELISPOT IFN-*γ*).

ND: not done.

**Table 2:** Serum IgG responses to seven mycobacterial antigens.

Group	Optical density at 450 nm (mean ± SD) and positive rate (%)						
	ESAT-6	CFP-10	MDP1	Ag85A	Acr	HBHA	HrpA
Cut-off values	0.067	0.077	0.118	0.037	0.129	0.140	0.072
Negative control	0.068 ± 0.155 36.4%	0.084 ± 0.160 41.9%	0.103 ± 0.108 36.5%	0.037 ± 0.074 44.6%	0.143 ± 0.213 32.4%	0.091 ± 0.157 23.0%	0.050 ± 0.210 16.2%
Active disease	0.248 ± 0.590^a^ 55.7%	0.252 ± 0.508^a^ 58.0%	0.157 ± 0.240 48.9%	0.068 ± 0.246 51.1%	0.487 ± 0.714^b^ 76.1%	0.351 ± 0.502^b^ 59.3%	0.256 ± 0.276^b^ 77.3%
Past disease	0.068 ± 0.129^c^ 39.3%	0.230 ± 0.345 64.3%	0.207 ± 0.259^d^ 54.8%	0.080 ± 0.136 56.0%	0.277 ± 0.381^c^ 66.7%	0.300 ± 0.436^d^ 58.3%	0.290 ± 0.264^e^ 79.8%
Latent TB infection	0.079 ± 0.100 41.2%	0.195 ± 0.190 76.5%	0.257 ± 0.320 52.9%	0.040 ± 0.115 41.2%	0.210 ± 0.292 64.7%	0.277 ± 0.334 64.7%	0.325 ± 0.368^f^ 82.4%

AUROC	0.63	0.59	0.52	0.51	0.74	0.74	0.82

^a^ *P* < 0.05 (active versus control), ^b^ *P* < 0.01 (active versus control), ^c^ *P* < 0.05 (active versus past), ^d^ *P* < 0.05 (past versus control), ^e^ *P* < 0.01 (past versus control), ^f^ *P* < 0.01 (latent versus control).

Optimal cut-off values were chosen when the Youden's index (sensitivity and specificity − 1) was maximal.

AUROC: areas under the receiver operating characteristic curve.

**Table 3:** Serum IgA responses to seven mycobacterial antigens.

Group	ELISA index at 450 nm (mean ± SD) and positive rate (%)						
	ESAT-6	CFP-10	MDP1	Ag85A	Acr	HBHA	HrpA
Cut-off values	1.496	0.567	1.115	0.577	0.738	1.015	0.901
Negative control	1.564 ± 1.533 32.4%	1.007 ± 1.041 58.1%	2.290 ± 2.277 63.5%	1.354 ± 1.382 64.9%	0.881 ± 0.739 41.9%	1.132 ± 1.140 35.1%	0.932 ± 0.813 39.2%
Active disease	2.189 ± 2.192 59.5%	1.246 ± 1.312 78.4%	1.137 ± 0.828^a^ 30.7%	1.470 ± 1.664 85.2%	1.252 ± 1.549^b^ 58.0%	1.713 ± 1.955 55.7%	0.751 ± 0.333 29.5%
Past disease	1.336 ± 1.128^c^ 23.8%	0.976 ± 0.715 72.6%	1.166 ± 1.232^d^ 34.5%	1.037 ± 0.686 79.8%	0.831 ± 0.495 50.0%	1.283 ± 0.954 50.0%	0.771 ± 0.356 35.7%
Latent TB infection	0.783 ± 0.246^e^ 0.0%	0.793 ± 0.307 64.7%	0.879 ± 0.297^f^ 11.8%	0.898 ± 0.395 58.8%	0.606 ± 0.221 23.5%	0.772 ± 0.299 17.6%	0.595 ± 0.285 17.6%

AUROC	0.63	0.53	0.66	0.57	0.59	0.62	0.53

^a^ *P* < 0.01 (active versus control), ^b^ *P* < 0.05 (active versus past), ^c^ *P* < 0.01 (active versus past), ^d^ *P* < 0.01 (past versus control), ^e^ *P* < 0.05 (active versus latent), ^f^ *P* < 0.05 (latent versus control).

Optimal cut-off values were chosen when the Youden's index (sensitivity and specificity − 1) was maximal.

AUROC: areas under the receiver operating characteristic curve.
