# Supplementary material for: Evaluation of Humoral Immunity to Mycobacterium tuberculosis-Specific Antigens for Correlation with Clinical Status and Effective Vaccine Development
Source: J Immunol Res. 2015 Oct 19;2015:527395. doi: 10.1155/2015/527395 (PMC4629042; doi:10.1155/2015/527395)
Supplement: Supplementary file 1 — Supplemental Table 1: Classification of various clinical laboratory test results that relate to the patients' general condition (serum albumin), inflammatory status (C-reactive protein level; CRP) and the disease status (smear at entry, positive conversion time, duration of culture negative, X-ray type and X-ray extent). Supplemental Table 2: List of the amount of recombinant Mtb antigens used to evaluate the serum IgG and IgA levels by ELISA as described in Subjects and Methods 2.5. Supplemental Table 3: Statistical analysis of the association between serum IgA levels and the levels of various clinical statuses classified in Supplemental Table 1. Supplemental Figure legends Supplemental Figure 1: Association between humoral IgG or IgA response and positivity of ELISPOT assays using the same antigens. There was no association between IgG or IgA values and ELISPOT positivity against the same antigens. Shaded areas: areas under cut-off values, vertical lines: mean values, +: ELISPOT positive and -: ELISPOT negative. Supplemental Figure 2: Association of QFT-IT assay values in active disease, past disease and LTBI patients. There was no significant association in these patients. Vertical lines: mean values, values: concentration of IFN-γ (IU/ml). Supplemental Figure 3: Association between humoral IgA responses and clinical scores measured by smear at entry. There was no association between IgA values and scores measured by smear at entry. Shaded areas: areas under cut-off values, vertical lines: mean values. Supplemental Figure 4: Association between humoral IgA responses and clinical scores measured by positive conversion time (weeks). There was no association between IgA values and scores measured by positive conversion time. Shaded areas: areas under cut-off values, vertical lines: mean values, ND: not done. Supplemental Figure 5: Association between humoral IgA responses and clinical scores measured by duration culture positive (days). There was no association betw [file 527395.f1.zip › 527395/tables.pdf]

Supplemental Table 1. Classification of Clinical Status

<table>
<tr><th></th><th colspan="3">(←minimal)</th><th colspan="3"></th><th colspan="3"></th><th colspan="3">(severe→)</th></tr>
<tr><td>Smear at entry [a]</td><td colspan="3">0 to ±</td><td colspan="3">1+</td><td colspan="3">2+</td><td colspan="3">3+</td></tr>
<tr><td>Positive conversion time (weeks) [b]</td><td colspan="3">≥ 3</td><td colspan="3">2</td><td colspan="3">1</td><td colspan="3">0</td></tr>
<tr><td>Duration of culture negative (days) [c]</td><td colspan="3">< 20</td><td colspan="3">20 ≤ <40</td><td colspan="3">40≤ < 60</td><td colspan="3">≥ 60</td></tr>
<tr><td>CRP at entry (mg/ml)</td><td colspan="6">< 2</td><td colspan="6">≥ 2</td></tr>
<tr><td>Albumin at entry (g/ml)</td><td colspan="4">≥ 4</td><td colspan="4">3 ≤ < 4</td><td colspan="4">< 3</td></tr>
<tr><td>CRP after 60 days (mg/ml)</td><td colspan="4">0</td><td colspan="4">< 2</td><td colspan="4">≥ 2</td></tr>
<tr><td>X-ray type (cavity) [d]</td><td colspan="4">III (no cavity)</td><td colspan="4">II (morbid foci than I)</td><td colspan="4">I (widespread)</td></tr>
<tr><td>X-ray extent [d]</td><td colspan="4">1</td><td colspan="4">2</td><td colspan="4">3</td></tr>
</table>

a: 0 (no acid fast bacilli (AFB) on smear), ± (1-2 AFB per 300 field), 1+ (1-9 AFB per 100 field), 2+ (more than 10 AFB per 100 field), and 3+ (more than 10 AFB per field).

b:Positive results (weeks) of MGIT from entry.

c: Durations (days) to be negative results of smear by sputum microscopy after initiation of the treatment.

d: “The Japanese Society for Tuberculosis Classification” was applied [20].

Supplemental Table 2. Amount of antigens used for the detection of serum IgG and IgA.

TB Antigens	IgG ELISA (μg/well)	IgA ELISA (μg/well)
ESAT-6 (Rv3875)	0.5	2.5
CFP-10 (Rv3874)	0.5	2.5
MDP1 (Rv2986c)	0.05	0.5
Ag85A (Rv3804c)	0.05	0.5
Acr (Rv2031c)	0.5	2.5
HBHA (Rv0475)	0.5	2.5
HrpA (Rv0251c)	0.5	2.5

Supplemental Table 3. Association between titers of IgA and clinical status.

		ESAT-6	CFP-10	MDP1	Ag85A	Acr	HBHA	HrpA
Smear at entry								
	r	0.154	-0.05877	-0.09966	0.026789	0.08973	0.05675	-0.03961
	p	0.19976	0.62637	0.40827	0.8245	0.45676	0.63828	0.74292
Positive Conversion time (weeks)								
	r	0.1018	0.01556	-0.02547	0.07465	0.04275	-0.00991	-0.07863
	p	0.40173	0.89829	0.83423	0.53912	0.72532	0.93511	0.51762
Duration culture negative (days)								
	r	0.0654	0.02624	0.06089	0.00791	0.05718	0.08827	-0.10502
	p	0.61353	0.83957	0.63824	0.95133	0.65893	0.4951	0.41661

CRP at entry (mg/ml)								
	r	0.10193	0.01868	0.02661	0.04139	0.1175	-0.11609	**-0.25058**
	p	0.39765	0.87709	0.82563	0.73183	0.32911	0.33502	**0.03506***
Albumin at entry (g/ml)								
	r	**0.33422**	0.05802	-0.03364	0.05185	**0.34487**	-0.04649	-0.0641
	p	**0.00439***	0.63078	0.78063	0.66761	**0.00323***	0.70025	0.59534
CRP after 60 days (mg/ml)								
	r	0.13127	0.11439	0.15752	0.10689	0.10689	0.19502	0.17252
	p	0.30514	0.37202	0.21759	0.40439	0.40439	0.12559	0.17635
X-ray type (cavity)								
	r	0.00668	-0.03311	0.0079	0.01562	-0.01287	0.05442	-0.02875
	p	0.9559	0.78397	0.94787	0.89711	0.91515	0.6522	0.81301
X-ray (extent)								

r	0.21033	0.01258	0.02114	0.13727	0.175153	0.05624	-0.08867
p	0.07831	0.91708	0.86112	0.25365	0.14402	0.64133	0.46212

Statistical significance ($p < 0.05$ or $p < 0.01$) appeared with an asterisk.

Supplemental Table 4. Association between TB gene and BCG ortholog (identity)

TB Gene	BCG ortholog (identity)
ESAT-6 (Rv3875)	Not identified
CFP-10 (Rv3874)	Not identified
MDP1 (Rv2986c)	BCG_3007 (0.958)
Ag85A (Rv3804c)	BCG_3866c (1.00)
Acr (Rv2031c)	BCG_2080 (1.00)
HBHA (Rv0475)	BCG_0516 (1.00)
HrpA (Rv0251c)	BCG_0257c (1.00)
