# Supplementary figures and images for: Evaluation of Humoral Immunity to Mycobacterium tuberculosis-Specific Antigens for Correlation with Clinical Status and Effective Vaccine Development
Source: J Immunol Res. 2015 Oct 19;2015:527395. doi: 10.1155/2015/527395 (PMC4629042; doi:10.1155/2015/527395)

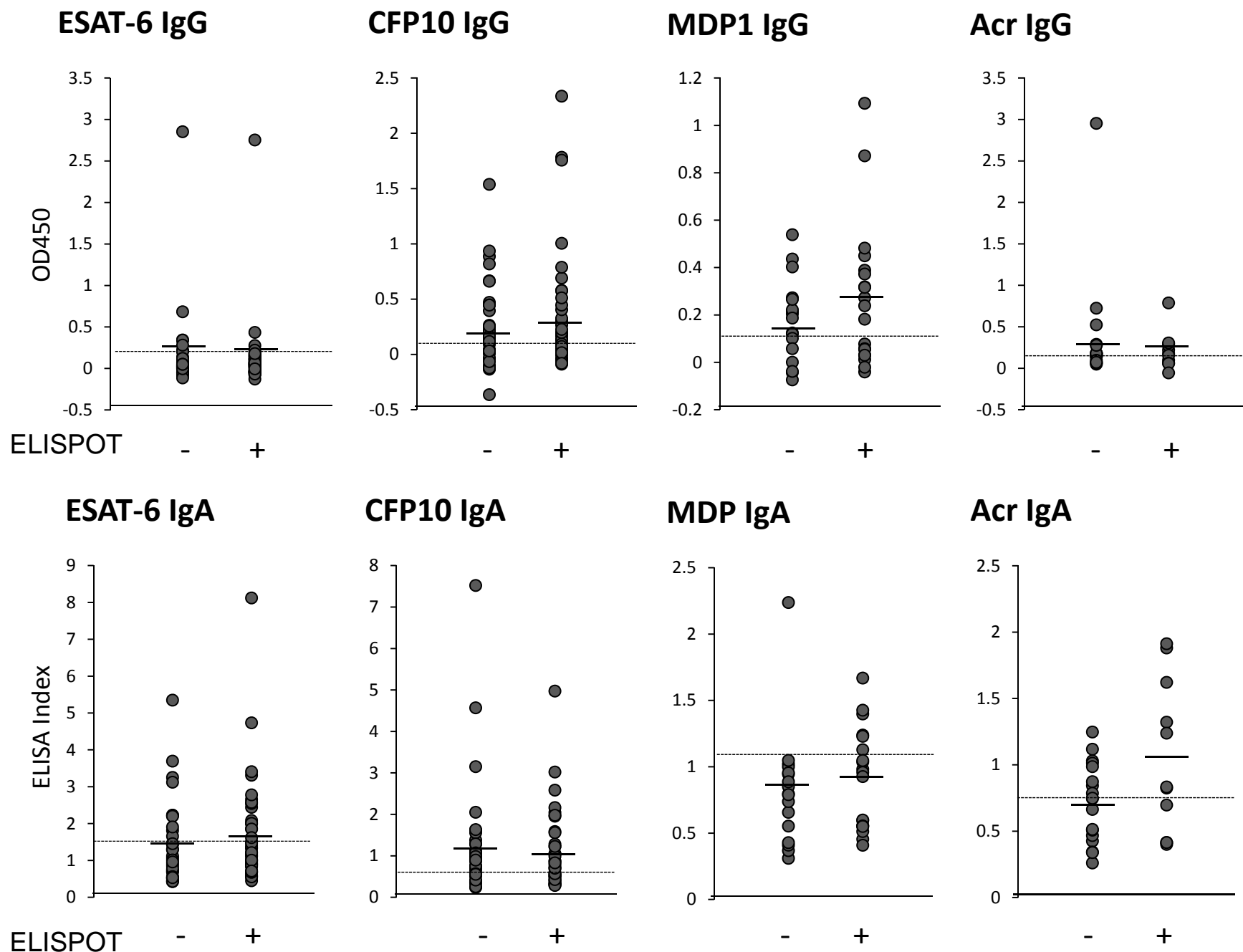

Supplemental Figure 1

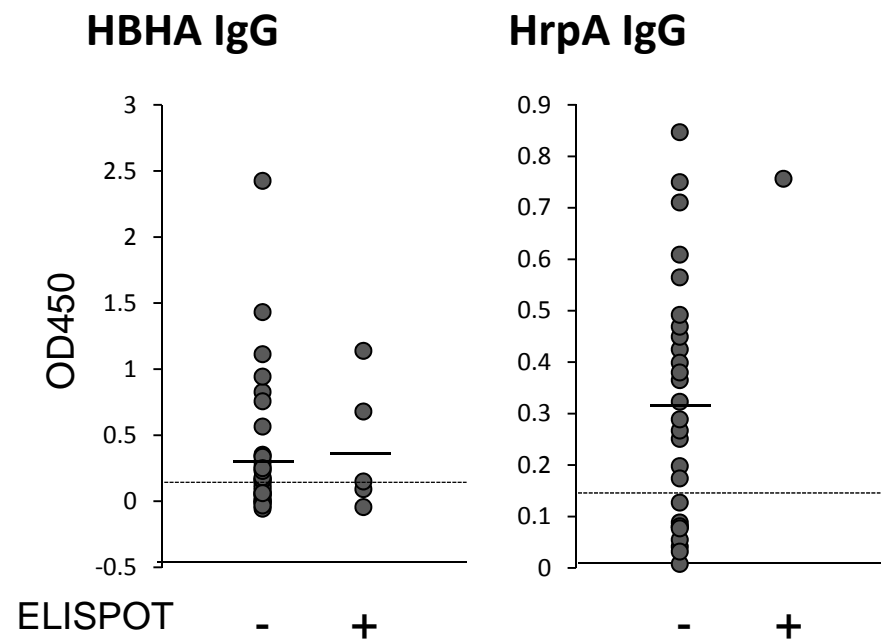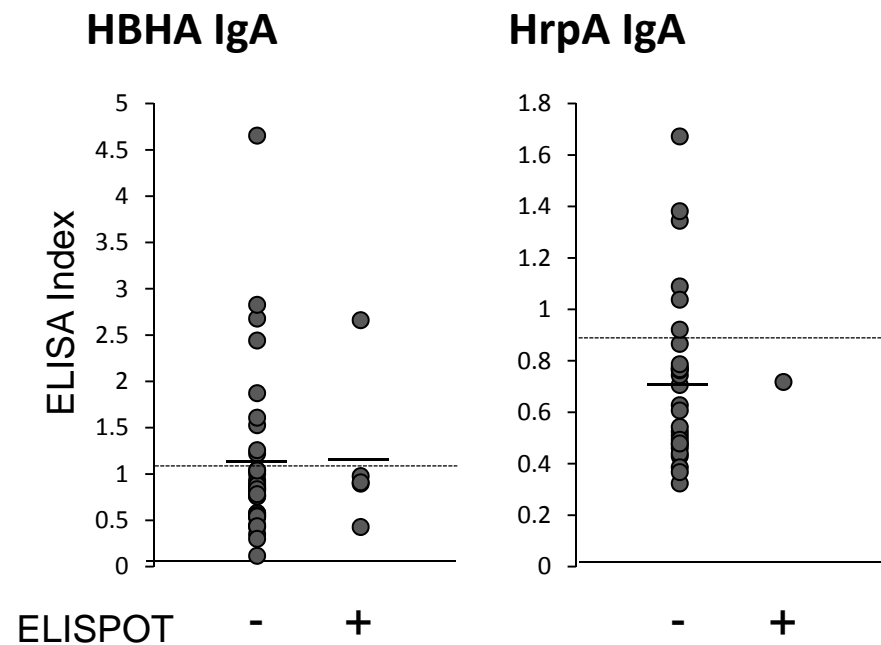

Supplemental Figure 1 (cont'd)

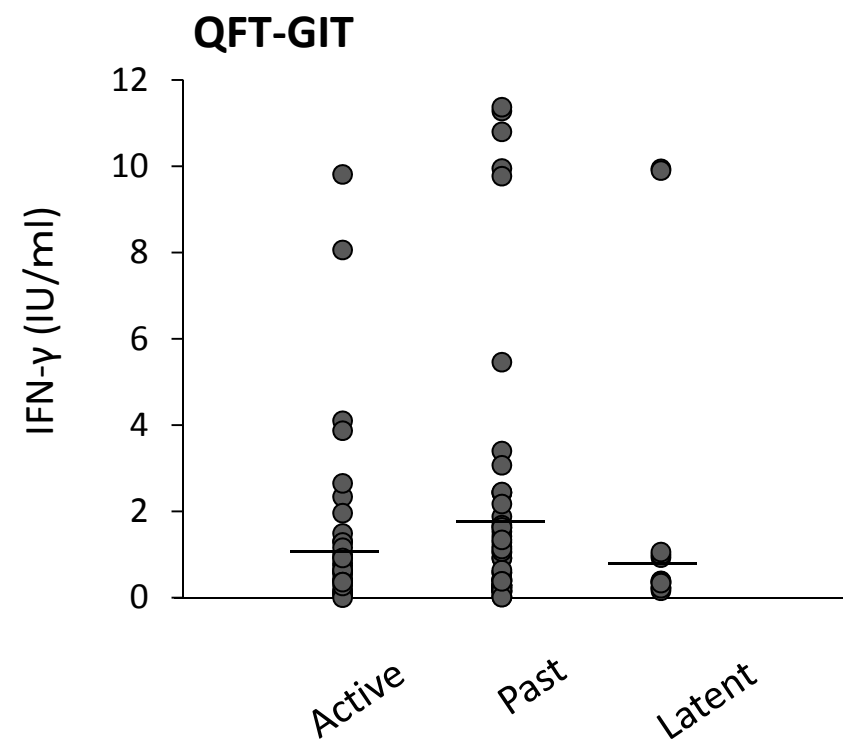

Supplemental Figure 2

Supplement: Supplementary file 1 — Supplemental Table 1: Classification of various clinical laboratory test results that relate to the patients' general condition (serum albumin), inflammatory status (C-reactive protein level; CRP) and the disease status (smear at entry, positive conversion time, duration of culture negative, X-ray type and X-ray extent). Supplemental Table 2: List of the amount of recombinant Mtb antigens used to evaluate the serum IgG and IgA levels by ELISA as described in Subjects and Methods 2.5. Supplemental Table 3: Statistical analysis of the association between serum IgA levels and the levels of various clinical statuses classified in Supplemental Table 1. Supplemental Figure legends Supplemental Figure 1: Association between humoral IgG or IgA response and positivity of ELISPOT assays using the same antigens. There was no association between IgG or IgA values and ELISPOT positivity against the same antigens. Shaded areas: areas under cut-off values, vertical lines: mean values, +: ELISPOT positive and -: ELISPOT negative. Supplemental Figure 2: Association of QFT-IT assay values in active disease, past disease and LTBI patients. There was no significant association in these patients. Vertical lines: mean values, values: concentration of IFN-γ (IU/ml). Supplemental Figure 3: Association between humoral IgA responses and clinical scores measured by smear at entry. There was no association between IgA values and scores measured by smear at entry. Shaded areas: areas under cut-off values, vertical lines: mean values. Supplemental Figure 4: Association between humoral IgA responses and clinical scores measured by positive conversion time (weeks). There was no association between IgA values and scores measured by positive conversion time. Shaded areas: areas under cut-off values, vertical lines: mean values, ND: not done. Supplemental Figure 5: Association between humoral IgA responses and clinical scores measured by duration culture positive (days). There was no association betw [file 527395.f1.zip › 527395/figs.pdf]
